# Interaction between coffee consumption and polygenic risk score in relation to diabetes: insights from the Maastricht study

**DOI:** 10.1007/s00394-025-03782-y

**Published:** 2025-08-20

**Authors:** Yufeng Rao, Evan Yi-Wen Yu, Tzu-Yao Lin, Yiming Chen, Yingfen Qin, Annemarie Koster, Simone J. P. M. Eussen, Hans Bosma, Tos T. J. M. Berendschot, Carla J. H. van der Kallen, Marleen M. J. van Greevenbroek, Bastiaan E. de Galan, Maurice P. Zeegers, Anke Wesselius

**Affiliations:** 1 Department of Epidemiology, Maastricht University, PO Box 616, 6200 MD Maastricht, The Netherlands; 2 Institute of Nutrition and Translational Research in Metabolism, Maastricht University, Maastricht, The Netherlands; 3 Key Laboratory of Environmental Medicine and Engineering of Ministry of Education, Department of Epidemiology & Biostatistics, School of Public Health, Southeast University, Nanjing, 210009 China; 4 Department of Methodology & Statistics, Maastricht University, Maastricht, The Netherlands; 5 Care and Public Health Research Institute (CAPHRI), Maastricht University, Maastricht, The Netherlands; 6 Quantitative Psychology and Individual Differences Research Group, Faculty of Psychology and Educational Sciences, KU Leuven, Leuven, Belgium; 7 Department of Endocrinology, The First Affiliated Hospital of GuangXi Medical University, Nanning, 530021 Guangxi People’s Republic of China; 8 Department of Social Medicine, Maastricht University, Maastricht, The Netherlands; 9 Cardiovascular Research Institute Maastricht (CARIM), Maastricht University, Maastricht, The Netherlands; 10 Mental Health and Neuroscience Research Institute (MeHNS), Maastricht University, Maastricht, The Netherlands; 11 University Eye Clinic Maastricht, Maastricht University Medical Center, Maastricht, The Netherlands; 12 Department of Internal Medicine, Maastricht University Medical Center+, Maastricht, The Netherlands; 13 Department of Internal Medicine, Radboud University Medical Center, Nijmegen, The Netherlands

**Keywords:** Polygenic risk score, Coffee consumption, Prediabetes, Type 2 diabetes mellitus, Dose–response

## Abstract

**Aims:**

This study investigated the associations of polygenic risk score (PRS) and coffee consumption, as well as their interaction, with prediabetes and type 2 Diabetes Mellitus (T2DM) among participants in the southern Netherlands.

**Methods:**

7668 participants were classified as normal glucose metabolism (NGM), prediabetes, or T2DM based on World Health Organization 2006 criteria. PRS (423 T2DM-related single nucleotide polymorphisms) and coffee consumption (via food frequency questionnaire) were categorized into tertiles (low, medium, and high) based on the population distribution. Multinomial logistic regression and dose–response analyses were performed to evaluate the cross-sectional associations between PRS and coffee consumption with prediabetes and T2DM.

**Results:**

Fully adjusted analyses indicated that medium and high coffee consumption were associated with lower odds of prediabetes (odds ratios [ORs]: 0.80; 95% CI: 0.69, 0.92 and 0.83; 95% CI: 0.72, 0.96) and T2DM (ORs: 0.80; 95% CI: 0.70, 0.91 and 0.80; 95% CI: 0.70, 0.91). U-shaped associations were observed for both prediabetes and T2DM, with the overlapping range of 2.9–6.9 cups/day statistically associated with lower odds (OR < 1) for both conditions. Additionally, participants in the PRS group had higher odds of prediabetes (OR: 1.58; 95% CI: 1.35, 1.86) and T2DM (OR: 3.16; 95% CI: 2.80, 3.56) compared to the low PRS group. No significant interaction was found between PRS and coffee consumption (*P* = 0.21).

**Conclusions:**

No significant interaction was observed between coffee consumption and PRS for prediabetes and T2DM. Coffee consumption was associated with the prevalence of both conditions in a pattern that may be U-shaped. However, these associations appear to be population-specific and require validation in diverse populations to clarify gene-lifestyle interactions.

**Supplementary Information:**

The online version contains supplementary material available at 10.1007/s00394-025-03782-y.

## Introduction

Diabetes represents a significant global health challenge, affecting millions worldwide. In 2021, approximately 537 million adults (aged 20–79 years) were living with diabetes, a number expected to rise to 783 million by 2045 [1]. Prediabetes, characterized by elevated blood glucose levels not yet in the diabetic range, also poses a critical concern, with a global prevalence of 14.9% (762 million) projected to increase to 16.5% (1052 million) by 2045 [2]. Without preventive measures, up to 50% of individuals with prediabetes may progress to diabetes within five years [3], leading to severe health complications and economic burdens on healthcare systems worldwide [4].

The development of diabetes is influenced by both genetic and lifestyle factors, underscoring the need for a multifaceted prevention strategy addressing inherent and modifiable risks. Genetic variations, as non-modifiable risk factors, have been linked to diabetes, with numerous single nucleotide polymorphisms (SNPs) identified as positively associated with the risk of type 2 diabetes mellitus (T2DM) [5–8]. The polygenic risk score (PRS), a composite measure of multiple genetic variants, has shown robust predictive power across diverse populations [9–11].

Lifestyle choices, including diet and physical activity, are modifiable factors that significantly influence T2DM prevention and management [12–15]. Diets with higher carbohydrate quality [16], nut consumption [17], and tea consumption [18] have been linked to lower T2DM incidence, while excessive sugar and starch consumption [19] and a lack of exercise [20] increase this risk. Habitual coffee consumption, a widely consumed beverage, has garnered attention for its potential protective effects against T2DM [21–23]. While prospective cohort studies suggest an inverse relationship between coffee consumption and T2DM risk, short-term randomized controlled trials present conflicting results, indicating increased postprandial blood glucose responses [24] and decreased insulin sensitivity [25] in certain populations.

The impact of coffee consumption on early glucose dysregulation, such as prediabetes, remains unclear [13, 26–30]. This is crucial, as intervening at the prediabetes stage could prevent progression to T2DM and alleviate its global burden. Although previous studies have examined the interaction between PRS and various lifestyle factors [31–36], but research specifically linking coffee consumption to prediabetes is limited. Only one study has explored coffee-PRS interactions, calculating PRS using a simple count of risk alleles [37], leaving questions about coffee’s role in preventing early glucose dysregulation unanswered.

While observational studies suggest that coffee consumption is associated with a lower risk of T2DM, it is uncertain whether this benefit extends to individuals at high genetic risk. Bioactive compounds in coffee, such as caffeine and antioxidants, may influence glucose metabolism and insulin sensitivity, potentially interacting with genetic pathways related to T2DM risk. We hypothesized that the impact of genetic predisposition on T2DM risk may be modified by habitual coffee consumption. Using data from The Maastricht Study, this study additionally focuses on prediabetes and aims to investigate the separate and interactive association of habitual coffee consumption and genetic susceptibility (as assessed by PRS) with the likelihood of prediabetes and T2DM.

## Methods and materials

### Study population

We used data from The Maastricht Study, an observational prospective population-based cohort study. The rationale and methodology have been described previously [38]. In brief, the study focuses on the etiology, pathophysiology, complications, and comorbidities of T2DM and is characterized by an extensive phenotyping approach. Eligible for participation were all participants aged between 40 and 75 years and living in the southern part of the Netherlands. Participants were recruited through mass media campaigns and from the municipal registries and the regional Diabetes Patient Registry via mailings. Recruitment was stratified according to known T2DM status, with an oversampling of participants with T2DM, for reasons of efficiency. The present report includes cross-sectional data from 9187 participants, who completed the baseline survey between November 2010 and October 2020. The examinations of each participant were performed within a time window of three months. The study has been approved by the institutional medical ethical committee (NL31329.068.10) and the Minister of Health, Welfare and Sports of the Netherlands (Permit 131088-105234-PG). All participants gave written informed consent. Exclusion criteria for the current study included only participants with type 1 diabetes (n = 49) and other types of diabetes (n = 4). Additionally, participants were excluded from the analysis due to unavailable data on coffee consumption (n = 580), genetic data (n = 707), or participants with implausible energy intake (<800 or >4200 kilocalories/day for men and <500 or >3500 kilocalories/day for women) were excluded (n = 179). As a result, a total of 7668 participants were included in this analysis (Supplementary Fig. 1).

### Assessment of prediabetes and diabetes

Participants fasted overnight and then underwent a 2-h oral glucose tolerance test (OGTT) with 75 g of glucose. Prediabetes and T2DM were identified using the World Health Organization 2006 criteria [39], which were standard at the time of data collection and remain widely used in the epidemiological studies. HbA1c was not included due to its unavailability for the full study sample. Prediabetes was defined as an FPG between 6.1 and 7.0 mmol/L or a 2-h post OGTT plasma glucose between 7.8 and 11.1 mmol/L. T2DM was diagnosed if participants met any of the following criteria: (1) a priori diagnosis by a physician or treatment with oral hypoglycemic medication or insulin; (2) a fasting plasma glucose (FPG) level of $\ge 7.0$ mmol/L; or (3) a 2-h plasma glucose level of $\ge 11.1$ mmol/L. Normal glucose metabolism (NGM) was defined as an FPG of $<6.1$ mmol/L and a 2-h plasma glucose level of $< 7.8$ mmol/L. Newly diagnosed diabetes was confirmed based on laboratory tests conducted at enrollment.

### Assessment of coffee consumption

All participants completed a validated 253-item food frequency questionnaire (FFQ) before being informed about their glucose metabolism status (e.g., NGM, prediabetes, or T2DM). The FFQ has been shown to have good overall validity [40]. Coffee consumption was calculated by multiplying the frequency of coffee consumption by the amount consumed, resulting in grams per day. In the Netherlands, the average serving size of coffee is 125 g per cup. To convert daily coffee consumption from grams to cups, the total grams consumed per day were divided by 125. This conversion improves interpretability and comparability with other studies, as cups per day is a more commonly used unit in nutritional research. Participants were then categorized into low, medium, and high coffee consumption groups based on the tertile distribution of coffee consumption within the study population.

### Assessment of PRS

#### DNA extraction, genotyping and quality control

In The Maastricht Study, genotyping was performed using the Illumina Global Screening Array BeadChip (Infinium iSelect 24 × 1 HTS Custom Beadchip Kit) at the Human Genotyping Facility of the Genetic Laboratory of the Department of Internal Medicine at Erasmus MC. Genotyping was successful for all included samples. Genotype data quality control (QC) and imputation were performed using the Rapid Imputation for COnsortias PipeLIne (RICOPILI) [41]. Preliminary QC in PLINK version 1.9 [42] (http://zzz.bwh.harvard.edu/plink/) consisted of checking for discrepancies between self-reported and genotype-based sex and dropping participants with non-identical sex, identifying relatedness or duplicate samples through identity-by-descent estimation, dropping strand-ambiguous SNPs, and dropping duplicate markers. Subsequent QC included multi-dimensional scaling to extract ancestry principal components (PCs), followed by extraction of individuals of European ancestry based on these PCs. Furthermore, genotype data was filtered based on the following criteria: DNA sample-level missingness (–mind 0.02), SNP-level missingness (–pre-geno 0.05; –geno 0.02), case–control missing rate difference (–midi 0.02), maximum number of Mendelian errors per SNP (–lmend 4), maximum number of Mendelian errors per sample (–imend 10,000), heterozygosity outliers based on the F statistic (–Fhet_th 0.2), violations of Hardy–Weinberg equilibrium (HWE) in controls and cases (–hwe_th_co 1 × 10^–6^; –hwe_th_ca 1 × 10^−10^), monomorphic SNPs (–withpna 0), and a minimum number of chromosome X SNPs required for sex check (–sexmin 10). Imputation through the RICOPILI consisted of (1) prephasing using Beagleagle v2.3.5 [42, 43] and (2) imputation using Minimac3 [44]. Genotype dosage data were converted to best guess genotypes using a minimum genotype probability threshold of *P* > 0.8.

The obtained genotypic data were aligned to the human reference genome (GRCh37/hg19), and PLINK was used for QC of the genetic data. We excluded duplicate SNPs and SNPs with a call rate < 90%. After further removing the genomic regions with long-range linkage disequilibrium (LD), including the major histocompatibility complex (MHC) region, we conducted principal component analysis (PCA) to identify individuals who deviated from the European population. In the PCA, we performed linkage disequilibrium (LD) pruning using PLINK for the remaining SNPs to identify independent variants with a window of 500 SNPs, a step size of 50 SNPs and an LD of r^2^ < 0.2. This step removed 33 individuals who were far deviated from European ancestry. After that, SNPs were mapped to the 1000 Genomes Project Phase 3 v5 by Beagle [45, 46], and then imputed with 1000 Genomes Project Phase 3 v5 reference panel by Minimac3 [44, 47]. We included SNPs with imputation accuracy; genetic association and differential expression (RSQR) >0.3, minor allele frequency (MAF) >0.01, HWE (*P* >1 × 10^−6^).

#### Selection and harmonization of SNPs for PRS

Initially, 425 SNPs were selected based on their genome-wide significant association with T2DM (*P* < 5 × 10^−8^) specifically in European populations, as reported in Supplementary Table 6 of the source multi-ancestry genome-wide association study (GWAS) [8]. These SNPs were identified through analysis of 228,499 participants with T2DM and 1,178,783 controls, with all of which passed quality control measures (call rate >0.975, HWE >1 × 10^−10^, INFO > 0.3, MAF > 0.1%). Subsequently, these SNPs were matched with those from The Maastricht Study, resulting in 423 SNPs used to calculate PRS for T2DM. Effect sizes of each SNP were derived from the aforementioned GWAS study [8], while genotype information was derived from The Maastricht Study.

#### Calculation of PRS

PRS is calculated using the following formula: $\text{PRS}={{\sum }_{i=1}^{n}\beta }_{i}{\text{SNP}}_{i}.$ Where $n$ is the total number of SNPs included, and ${\beta }_{i}$ is the effect size of each $SN{P}_{i}$ from the GWAS study [8]. The genotype for each $SN{P}_{i}$ is coded as $0, 1 or 2,$ corresponding to non-carriers, heterozygous carriers, and homozygous carriers of the effect allele, respectively, based on data from The Maastricht Study. Participants were then categorized into low, medium, and high genetic risk groups based on the tertile distribution of the PRS within the study population.

### Assessment of covariates

Covariates included age (years, continuous), sex (male or female), body mass index (BMI, kg/m^2^, continuous), FFQ based alcohol (gram/day, continuous) and energy intake [40] (kcal/day, continuous), Dutch Healthy Diet (DHD, continues) [48], sugar consumption (gram/day, continuous), smoking status (never, current, or former smoker), number of steps while awake (minutes/day, continuous), cardiovascular disease (CVD, yes or no), serum total cholesterol (mmol/l, continuous), mean arterial pressure (mmHg, continuous), use of blood pressure lowering medication (yes or no), use of lipid-modification medication (yes or no), family history of diabetes (yes, no, or I don’t know) and educational level (low, medium, or high). These covariates were obtained from physical examination, laboratory assessment, FFQ and medication interview.

### Statistical analysis

Normality of continuous variables was assessed using the Shapiro–Wilk test, and none were normally distributed. Therefore, continuous variables were presented as medians with interquartile range (IQR), and categorical variables were presented as numbers and percentages (%). To compare differences between NGM, prediabetes and T2DM, the following statistical tests were used: Kruskal–Wallis tests for continuous variables; and the chi-square tests for categorial variables.

Multinomial logistic regression analyses were conducted to assess the association of PRS and coffee consumption with diabetes status (i.e. NGM vs. prediabetes, NGM vs. T2DM). These analyses were performed using multinom () function from the nnet package in R (version 4.3.1) and produced odds ratios (OR) with 95% confidence intervals (CI). In these analyses, the low PRS group and the low coffee consumption group served as reference groups. The models were adjusted for confounders in three steps. Model 1 adjusted for age, sex, and educational level. Model 2 further adjusted for body mass index (BMI), alcohol consumption, smoking status, number of steps while awake, Dutch Healthy Diet (DHD), energy intake. Model 3 included additional adjustments for cardiovascular disease (CVD), serum total cholesterol, mean arterial pressure, use of blood pressure lowering medication, use of lipid-modification medication, sugar consumption, and family history of diabetes. Finally, the dose response association between coffee consumption (measured in cups per day) and diabetes prevalence was confirmed using restricted cubic spline function, with four knots placed at the 5th, 35th, 65th, and 95th percentiles.

To investigate whether coffee consumption modified the association between the PRS and diabetes, we used a likelihood ratio test to evaluate the statistical significance of the multiplicative interaction term (coffee * PRS) in model 3, treating both coffee and PRS as categorical variables. Additionally, a stratified analysis based on coffee consumption (low, medium, and high) was performed to assess the joint association between the coffee consumption and diabetes prevalence across groups with different levels of genetic susceptibility to diabetes.

To ensure the robustness of the findings, two sensitivity analyses were conducted. First, binary logistic regression was applied to participants with newly diagnosed diabetes, excluding those with a priori diagnosis to minimize bias. Second, to account for potential temporal variability related to the extended recruitment period (2010–2020), a binary variable representing early (2010–2014) vs. late (2015–2020) recruitment was included as a covariate in the adjusted models.

All statistical analyses were performed using R statistical software, version 4.3.1 [49]. Genotypic data QC and processing were conducted using multiple specialized tools. Preliminary QC, including sex-check, identification of duplicate samples, and variant filtering, was performed using PLINK version 1.9 [42]. Genotype pre-phasing was carried out with Beagle v2.3.5 [42, 43], followed by genotype imputation using Minimac3 [44] with the 1000 Genomes Project Phase 3 v5 reference panel [44, 47]. The entire pipeline was implemented through RICOPILI [41] to ensure consistency and reproducibility. PCA and LD pruning were also performed using PLINK 1.9 [42] to correct for population stratification and identify independent variants. The restricted cubic spline function was implemented using the R package: plotRCS [50]. A two-sided *P* value of <0.05 was considered statistically significant.

## Results

### Characteristics of participants with different glucose metabolism status

Overall, 7668 participants were included in this study, with a median age of 61 years, and 49.7% (3810) were male. Among them, 1641 (21.4%) were diagnosed with T2DM (including 325 newly diagnosed diabetes), 1159 (15.1%) had prediabetes, and 4868 (63.5%) had NGM.

All potential confounders differed significantly across the NGM, prediabetes, and T2DM groups, except for coffee consumption and energy intake (*P* > 0.05) (Table 1). Comparing NGM to prediabetes and T2DM, individuals with T2DM were older, had a lower number of steps while awake, and were more likely to be smokers. Additionally, they had lower educational attainment, higher BMI, and a higher prevalence of family history of diabetes. Interestingly, those with prediabetes showed an increasing PRS as they progressed toward T2DM (Table 1).

**Table 1:** Characteristics of study participants according to glucose tolerance status^a^

Characteristics	Total	NGM	Prediabetes	T2DM	*P*^†^
	N = 7668	N = 4868	N = 1159	N = 1641	
Sex					< 0.001
Male	3810 (49.69%)	2083 (42.79%)	622 (53.67%)	1105 (67.34%)	
Female	3858 (50.31%)	2785 (57.21%)	537 (46.33%)	536 (32.66%)	
Age (years)	61.00 [54.00, 67.00]	59.00 [52.00, 65.00]	64.00 [58.00, 68.00]	65.00 [58.00, 69.00]	< 0.001
Education level (%):					< 0.001
Low	2511 (33.18%)	1319 (27.37%)	439 (38.54%)	753 (46.77%)	
Medium	2073 (27.39%)	1348 (27.97%)	298 (26.16%)	427 (26.52%)	
High	2984 (39.43%)	2152 (44.66%)	402 (35.29%)	430 (26.71%)	
Coffee consumption (cups/day)	3.35 [2.00, 5.00]	3.44 [2.00, 5.00]	3.00 [2.00, 5.00]	3.90 [2.00, 5.20]	0.197
PRS	2.15 [1.60, 2.72]	2.03 [1.48, 2.61]	2.23 [1.65, 2.79]	2.46 [1.97, 2.92]	< 0.001
Number of steps while awake (minutes/day)	116.32 [90.72, 144.69]	121.80 [97.50, 149.86]	113.51 [88.03, 140.20]	98.92 [72.65, 127.23]	< 0.001
BMI (kg/m^2^)	26.20 [23.80, 29.10]	25.10 [23.00, 27.50]	27.40 [25.00, 30.10]	29.40 [26.40, 32.60]	< 0.001
Mean arterial pressure (mmHg)	94.00 [87.00, 102.00]	92.00 [85.00, 100.00]	97.00 [90.00, 104.00]	98.00 [91.00, 105.00]	< 0.001
Sugar consumption (gram/day)	0.00 [0.00, 2.15]	0.00 [0.00, 2.50]	0.00 [0.00, 2.85]	0.00 [0.00, 0.10]	< 0.001
Energy intake (kcal/day)	2048.51 [1691.53, 2491.85]	2054.47 [1699.56, 2503.95]	2059.65 [1682.52, 2492.20]	2020.35 [1673.37, 2445.23]	0.193
Alcohol total (gram/day)	8.10 [1.60, 18.01]	8.42 [2.08, 17.61]	9.64 [1.75, 20.20]	5.60 [0.52, 16.53]	< 0.001
DHD (range 0–140)	84.30 [73.60, 94.91]	85.99 [75.28, 96.45]	82.66 [72.01, 93.59]	80.43 [70.19, 90.05]	< 0.001
HDL (mmol/l)	1.50 [1.20, 1.80]	1.60 [1.30, 1.90]	1.40 [1.20, 1.70]	1.20 [1.00, 1.50]	< 0.001
LDL (mmol/l)	3.00 [2.30, 3.70]	3.20 [2.60, 3.80]	3.20 [2.50, 3.90]	2.30 [1.80, 2.90]	< 0.001
Total cholesterol (mmol/l)	5.20 [4.40, 6.00]	5.40 [4.70, 6.10]	5.30 [4.60, 6.10]	4.30 [3.70, 5.10]	< 0.001
Triglycerides (mmol/l)	1.19 [0.87, 1.67]	1.06 [0.80, 1.45]	1.40 [1.03, 1.90]	1.54 [1.10, 2.13]	< 0.001
Blood pressure lowering medication (n%)	2757 (35.99%)	1074 (22.09%)	520 (44.91%)	1163 (70.91%)	< 0.001
Lipid-modifying medication (n%)	2271 (29.64%)	727 (14.95%)	376 (32.47%)	1168 (71.22%)	< 0.001
Smoking (n%)					< 0.001
Never	2911 (38.24%)	2033 (41.97%)	393 (34.17%)	485 (29.98%)	
Former	3764 (49.45%)	2240 (46.24%)	626 (54.43%)	898 (55.50%)	
Current	937 (12.31%)	571 (11.79%)	131 (11.39%)	235 (14.52%)	
CVD (%)	1240 (16.35%)	591 (12.26%)	204 (17.75%)	445 (27.62%)	< 0.001
Family history of diabetes (n%)					< 0.001
Yes	2417 (31.92%)	1145 (23.73%)	367 (32.05%)	905 (56.56%)	
No	4082 (53.92%)	3012 (62.41%)	602 (52.58%)	468 (29.25%)	
I don’t know	1072 (14.16%)	669 (13.86%)	176 (15.37%)	227 (14.19%)	

NGM, normal glucose metabolism; T2DM, type 2 diabetes mellitus; BMI, body mass index; kcal, kilocalories; DHD, Dutch Healthy Diet; HDL, Serum HDL cholesterol; LDL, Serum LDL cholesterol; CVD, cardiovascular disease; PRS, polygenic risk score

*P* < 0.05 was considered statistically significant

^†^*P* values were calculated by the Kruskal–Wallis tests for continuous variables, or chi-squared test for categorial variables

^a^The demographic and clinical characteristics were expressed with as medians [Q1, Q3], or numbers (%)

### Coffee consumption, PRS and prediabetes/T2DM

In the fully adjusted model, higher coffee consumption was associated with lower odds of both prediabetes and T2DM. Compared to the low coffee consumption group, participants in the medium and high coffee consumption groups showed ORs of 0.80 (95% CI: 0.69, 0.92) and 0.83 (95% CI: 0.72, 0.96), respectively, for having prediabetes. A similar trend was observed for T2DM, with ORs of 0.80 (95% CI: 0.70, 0.91) and 0.80 (95% CI: 0.70, 0.91) for the medium and high coffee consumption groups, respectively (Table 2). PRS was positively associated with prediabetes and T2DM in the fully adjusted model. Compared to the low PRS group, participants in the medium PRS group had ORs of 1.36 (95% CI: 1.16, 1.59) for having prediabetes and 2.28 (95% CI: 2.02, 2.57) for having T2DM. In the high PRS group, the ORs were 1.58 (95% CI: 1.35, 1.86) for prediabetes and 3.16 (95% CI: 2.80, 3.56) for T2DM (Table 2).

**Table 2:** Association between coffee consumption, PRS and the prevalence of prediabetes/T2DM (n = 7668; OR (95% CI))

Predictor		Prediabetes			T2DM		
		Model 1	Model 2	Model 3	Model 1	Model 2	Model 3
Coffee	Low	1	1	1	1	1	1
	Medium	0.76 (0.65, 0.90)	0.80 (0.70, 0.92)	0.80 (0.69, 0.92)	0.80 (0.69, 0.93)	0.83 (0.73, 0.95)	0.80 (0.70, 0.91)
	High	0.89 (0.76, 1.04)	0.85 (0.74, 0.97)	0.83 (0.72, 0.96)	0.97 (0.84, 1.13)	0.89 (0.78, 1.01)	0.80 (0.70, 0.91)
PRS	Low	1	1	1	1	1	1
	Medium	1.30 (1.10, 1.53)	1.41 (1.21, 1.63)	1.36 (1.16, 1.59)	2.32 (1.97, 2.74)	2.53 (2.25, 2.85)	2.28 (2.02, 2.57)
	High	1.69 (1.43, 1.98)	1.73 (1.50, 2.00)	1.58 (1.35, 1.86)	3.65 (3.11, 4.29)	4.11 (3.66, 4.62)	3.16 (2.80, 3.56)

Model 1: was adjusted for age, sex, and educational level

Model 2: was additionally adjusted for body mass index (BMI), alcohol consumption, smoking status, number of steps while awake, Dutch Healthy Diet (DHD), and energy intake

Model 3: was additionally adjusted for cardiovascular disease (CVD), serum total cholesterol, mean arterial pressure, use of blood pressure lowering medication, use of lipid-modification medication, sugar consumption, and family history of diabetes

Statistical data for different coffee groups (median [Q1, Q3], min–max): Low coffee group = 2.0 [0.7, 2.2], 0–2.6; Medium coffee group = 3.9 [3.0, 4.0], 2.8–4.0; High coffee group = 6.0 [5.2, 7.0], 4.3–16.9

Statistical data for different PRS groups (median [Q1, Q3], min–max): Low PRS group = 1.4 [1.0, 1.6], -0.9–1.8; Medium PRS group = 2.2 [2.0, 2.3], 1.8–2.5; High PRS group = 3.0 [2.7, 3.3], 2.5–4.9

**Fig. 1:**
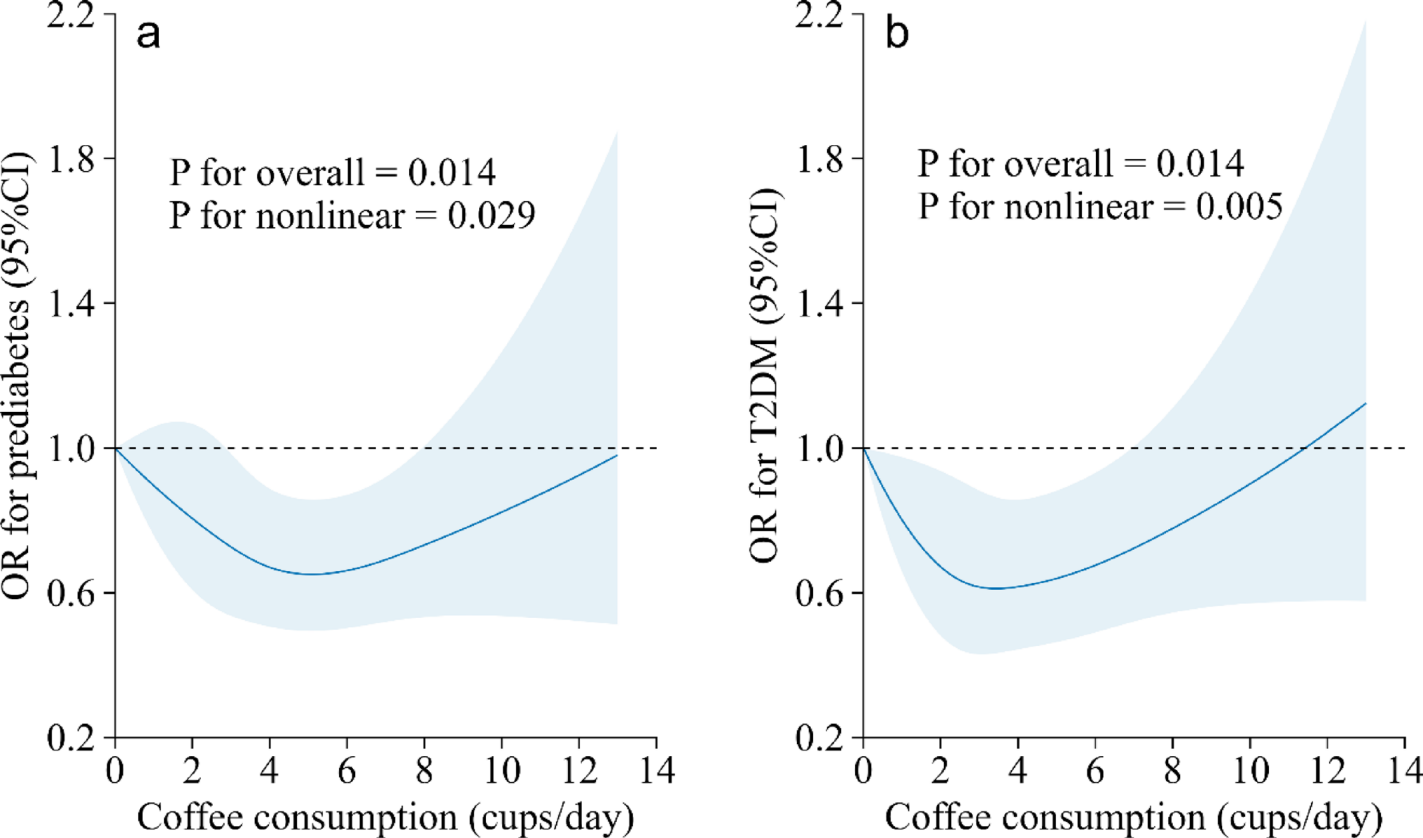
Dose–response association between coffee consumption and the prevalence of prediabetes (n = 6027) and T2DM (n = 6509). The dose–response associations between coffee consumption and the prevalence of prediabetes (panel a) and T2DM (panel b) were modeled using restricted cubic splines with 4 knots placed at the 5th, 35th, 65th, 95th percentiles of coffee consumption. The reference was set at the 5th percentile, corresponding to approximately 0 cups/day in both panels. Models were adjusted age, sex, educational level, body mass index (BMI), alcohol consumption, smoking status, number of steps while awake, Dutch Healthy Diet (DHD), energy intake, cardiovascular disease (CVD), serum total cholesterol, mean arterial pressure, use of blood pressure lowering medication, use of lipid-modification medication, sugar consumption, and family history of diabetes. The solid blue line indicates ORs, the blue shaded area indicates 95% Cis. OR, odds ratio; CI, confidence interval

However, the dose–response analysis revealed a U-shaped relationship between coffee consumption and the odds of prediabetes and T2DM. As coffee consumption increased, the prevalence of both prediabetes and T2DM progressively decreased, reaching minimum values at approximately 5.1 and 3.5 cups/day, respectively, after which they tended to increase. The overlapping range of 2.9–6.9 cups/day was associated with significantly lower odds for both conditions (Fig. 1).

### The interaction between PRS and coffee consumption with prediabetes/T2DM

The likelihood ratio test for the multiplicative interaction term (coffee * PRS), based on the multinomial logistic regression (Model 3), did not show statistical significance (*P* = 0.21), suggesting no evidence of interaction between coffee consumption and PRS across different glucose metabolism status (NGM, prediabetes, T2DM). Furthermore, the stratified analysis showed no significant differences in the association between PRS and the odds of either prediabetes or diabetes across various coffee consumption categories (Table 3).

**Table 3:** The interaction between PRS and coffee consumption and the prevalence of prediabetes/T2DM (OR (95% CI))

Coffee	PRS	Prediabetes			T2DM		
		Model 1	Model 2	Model 3	Model 1	Model 2	Model 3
Low (n = 2949)	Low	1	1	1	1	1	1
	Medium	1.24 (0.96, 1.61)	1.35 (1.07, 1.71)	1.33 (1.02, 1.73)	2.06 (1.58, 2.67)	2.19 (1.80, 2.66)	2.39 (1.97, 2.91)
	High	1.73 (1.34, 2.23)	1.75 (1.39, 2.21)	1.62 (1.25, 2.10)	2.95 (2.28, 3.82)	3.28 (2.70, 3.98)	2.77 (2.28, 3.37)
Medium (n = 2369)	Low	1	1	1	1	1	1
	Medium	1.50 (1.11, 2.02)	1.61 (1.23, 2.11)	1.58 (1.18, 2.12)	2.66 (1.96, 3.61)	2.95 (2.38, 3.65)	2.28 (1.84, 2.82)
	High	2.01 (1.49, 2.73)	2.31 (1.76, 3.01)	2.10 (1.56, 2.82)	4.82 (3.56, 6.51)	6.12 (4.98, 7.53)	4.47 (3.62, 5.52)
High (n = 2350)	Low	1	1	1	1	1	1
	Medium	1.18 (0.88, 1.58)	1.28 (0.99, 1.64)	1.22 (0.92, 1.63)	2.37 (1.78, 3.18)	2.64 (2.14, 3.25)	2.31 (1.86, 2.87)
	High	1.36 (1.01, 1.83)	1.29 (1.00, 1.67)	1.17 (0.87, 1.56)	3.65 (2.75, 4.86)	3.81 (3.10, 4.67)	2.81 (2.26, 3.49)

Model 1: was adjusted for age, sex, and educational level

Model 2: was additionally adjusted for body mass index (BMI), alcohol consumption, smoking status, number of steps while awake, Dutch Healthy Diet (DHD), and energy intake

Model 3: was additionally adjusted for cardiovascular disease (CVD), serum total cholesterol, mean arterial pressure, use of blood pressure lowering medication, use of lipid-modification medication, sugar consumption, and family history of diabetes

Statistical data for different coffee groups (median [Q1, Q3], min–max): Low coffee group = 2.0 [0.7, 2.2], 0–2.6; Medium coffee group = 3.9 [3.0, 4.0], 2.8–4.0; High coffee group = 6.0 [5.2, 7.0], 4.3–16.9

Statistical data for different PRS groups (median [Q1, Q3], min–max): Low PRS group = 1.4 [1.0, 1.6], −0.9 to 1.8; Medium PRS group = 2.2 [2.0, 2.3], 1.8–2.5; High PRS group = 3.0 [2.7, 3.3], 2.5–4.9

### Sensitivity analyses

#### Coffee consumption, PRS and newly diagnosed diabetes

Among individuals with newly diagnosed diabetes, a similar pattern in the association between coffee consumption and T2DM was observed. Compared to the low coffee consumption group, the ORs were 0.88 (95% CI: 0.63, 1.23) in the medium consumption group and 0.76 (95% CI: 0.53, 1.10) in the high consumption group. However, these results did not reach statistical significance (Supplementary Table 1). In the same population, PRS was positively associated with newly diagnosed diabetes, with ORs of 1.50 (95% CI: 1.05, 2.15) in the medium PRS group and 1.99 (95% CI: 1.40, 2.83) in the high PRS group compared with the low PRS group (Supplementary Table 1).

The dose–response analysis also revealed a U-shaped association between coffee consumption and the odds of newly diagnosed diabetes. Within the range of 0.065–9.1 cups/day, coffee consumption was associated with significantly lower odds compared to the reference. At the boundaries of this range, the odds were estimated at 0.97 (95% CI: 0.95, 0.99) and 0.50 (95% CI: 0.25, 0.99), with the lowest point observed at 2.9 cups/day (OR: 0.43, 95% CI: 0.25, 0.73). Beyond this interval, the odds began to rise again, supporting the observed non-liner trend (Supplementary Fig. 2).

Similarly, for newly diagnosed diabetes, no interaction was observed (*P* = 0.47), reinforcing these findings (Supplementary Table 2).

#### Coffee consumption, PRS and prediabetes/T2DM adjusted for recruitment year

To assess the potential impact of the extended recruitment period, we included a binary variable for early (2010–2014) versus late (2015–2020) recruitment as a covariate in the adjusted models. The results remained materially unchanged, indicating that the associations observed between coffee consumption, PRS and the prevalence of prediabetes and T2DM were robust to adjustment for recruitment timing (Supplementary Table 3).

## Discussion

This study aimed to investigate the independent associations of habitual coffee consumption and genetic susceptibility, as assessed by PRS, with the prevalence of prediabetes and T2DM among participants in the southern Netherlands. The analyses revealed that coffee consumption of 2.9–6.9 cups/day was associated with the lowest odds of both prediabetes and T2DM. Notably, coffee consumption above this range was associated with higher odds of these conditions, suggesting a potential U-shaped association. Additionally, participants with a higher PRS had a greater likelihood of having prediabetes and T2DM. However, there was no significant effect modification by coffee consumption on the relationship between PRS and diabetes risk.

Our study results align with previous studies [11, 51] that have highlighted the significant role of genetics in diabetes prevalence, showing that participants with a higher PRS face a greater likelihood of developing the condition. Specifically, each standard deviation increase in PRS was associated with a 23% higher odds of prediabetes and a 43% higher odds of T2DM at one-year follow-up [10]. This finding held true across different ancestries, as evidenced by PRS validation in Hispanic/Latino cohorts [10]. These results suggest that PRS can improve risk stratification, screening, and disease management across diverse populations.

Consistent with earlier observational studies and meta-analyses [23, 26, 52, 53], our findings show that optimal coffee consumption (from 2.9 to 6.9 cups/day) is associated with a lower prevalence of prediabetes and T2DM in this population. Notably, when coffee consumption exceeded this range, the odds of prediabetes and T2DM appeared to increase, suggesting a potential U-shaped association. However, these findings should not be generalized as universally protective. Overall, the data highlight a possible link between coffee consumption and lower prevalence in the early stages of dysglycemia. However, the underlying mechanisms behind these associations remain complex and not fully understood, with mixed research findings. Although caffeine as a prominent ingredient in coffee can acutely increase glucose and reduce insulin sensitivity [54], this effect disappears with chronic exposition [55]. More importantly, previous studies have reported that both decaffeinated and caffeinated coffee consumption are associated with lower risks of diabetes [56, 57], suggesting that components other than caffeine may contribute to the potential protective associations observed in those studies. Bioactive components such as chlorogenic acids, melanoidins, trigonelline, and the diterpenes cafestol and kahweol have been proposed as potential contributors to coffee’s metabolic effects [58]. These compounds may exert antioxidant, anti-inflammatory, or insulin-sensitizing effects, which could underlie the associations we observed between coffee intake and lower odds of prediabetes and T2DM. However, the concentration of these compounds can vary by coffee type and preparation method. Additionally, similar bioactives, particularly polyphenols and chlorogenic acids, are present in a variety of other foods, including fruits and vegetables, which were not individually quantified in our analysis [59]. As such, we cannot exclude residual confounding by other dietary sources of these compounds. Further research using more detailed dietary data or biomarker-based approaches is needed to clarify the mechanisms and specificity of these associations.

In recent years, researchers have focused on whether healthy lifestyle factors, including diet, can mitigate genetic susceptibility to diabetes. While some studies [31–36] have suggested that dietary patterns can modify the relationship between genetic risk and T2DM, others [60–62] have found no significant interaction between lifestyle factors and genetic susceptibility. These mixed findings may be attributed to limited SNPs and the omission of beta effect for each SNP, both of which can lower statistical power and reduce the ability to detect true associations. Many studies constructed PRS using a limited number of SNPs [34, 35, 60, 61], which may not accurately reflect overall genetic risk. Alternatively, some studies have calculated PRS by simply summing the number of risk alleles [31–33, 37] for each SNP rather than incorporating the beta effect obtained from the natural logarithm of the odds ratio reported in GWAS, which may further diminish statistical power. In contrast, our study utilized 423 T2DM associated SNPs from the largest GWAS to date, incorporating the beta effect of each SNP to more precisely quantify genetic risk [8].

Despite these advances, our analysis did not identify a statistically significant interaction between coffee consumption and the PRS constructed for T2DM-related SNPs. One possible explanation is that the PRS used in our study was limited to genetic variants directly associated with T2DM risk, without incorporating variants related to coffee or caffeine metabolism. Supporting this, previous studies have examined the interaction of coffee consumption with SNPs related to various metabolic pathways, including those specifically related to coffee metabolism, and found significant interactions. For example, one study investigated the interaction between coffee consumption and incretin-related SNPs, finding an inverse association with T2DM risk among carriers of specific risk alleles [37]. Similarly, another study found that high coffee consumption among individuals in the top PRS group (based on coffee metabolism SNPs) was associated with lower odds of prediabetes and diabetes [63].

These findings suggest that SNPs related to coffee metabolism, rather than solely diabetes-focused ones, may better capture gene-coffee interactions relevant to diabetes. Additionally, other studies have highlighted variations, such as CYP1A2, that influence caffeine metabolism rates and plasma glucose levels [64, 65], which may help clarify the role of caffeine-linked pathways. Although some of these SNPs are related to glucose metabolism, they were not included in our PRS selection, which was based on a large European GWAS sample and may reflect population-specific discrepancies [8]. This limitation may have contributed to the absence of detected interaction effects in our analysis.

Nevertheless, our results suggest the genetic predisposition to diabetes remains relatively consistent, independent of environmental exposures, possibly reflecting the multifaceted genetic architecture of T2DM. Future studies incorporating both T2DM-associated SNPs and variants involved in caffeine metabolism pathways will be important for further clarifying the relationships among coffee consumption, genetic variation, and diabetes risk.

### Study strengths and limitations

#### Strengths

Our study has several notable strengths. PRS was carefully calculated using 423 diabetes-related SNPs from the latest gene-wide association analysis [8], providing a strong genetic basis for our analysis.

The large sample size enhances the statistical power and reliability of our findings, allowing for a more robust generalization to the broader population. The population-based design, along with the strategic oversampling of participants with T2DM, facilitates precise comparisons between individuals with T2DM, prediabetes, and NGM.

This extensive design enabled us to distinctly consider prediabetes and employ thorough phenotyping, providing a detailed understanding of the relationships under investigation. These methodological strengths collectively improve the validity and applicability of our findings in diabetes research.

#### Limitations

Our study is not without limitations. First, the cross-sectional design prevents the assessment of temporal or causal relationships. However, participants completed the FFQ before learning their glucose metabolism status, which helps minimize the risk of reporting bias. Additionally, excluding participants previously diagnosed with T2DM from the analysis produced similar associations, suggesting that our findings are robust. It is important to acknowledge that reverse causation cannot be ruled out, as individuals diagnosed with prediabetes or T2DM might alter their coffee consumption, for example, by reducing consumption due to health concerns. Such behavior could bias the observed associations and partly explain the U-shaped relationship found. Therefore, our results should be interpreted with caution, and prospective longitudinal studies are needed to confirm the direction and causality of these associations.

Second, the diagnostic criteria for prediabetes and diabetes were based on the WHO 2006 guidelines, as HbA1c measurements were unavailable for the full study population. Although more recent standards, such as the 2025 ADA criteria, include HbA1c for classification, applying these retrospectively was not feasible. While the WHO 2006 criteria remain widely used and valid in epidemiological research, their use may limit direct comparability with studies adopting updated diagnostic thresholds.

Third, participants with known T2DM, self-reported coffee consumption may be especially susceptible to recall bias and misclassification, as they might modify or inaccurately report their consumption due to a heightened awareness of their condition and its dietary implications. Nevertheless, FFQs remain the most commonly used method to assess food intake in large cohorts and effectively rank participants by intake levels. The impact of this bias appears minimal, as similar associations were observed in both participants with newly diagnosed and known T2DM in our study.

Fourth, the FFQ questionnaire used in this study could not differentiate between the types of coffee consumed or whether it contained caffeine. However, existing research indicates that both caffeinated and decaffeinated coffee, as well as caffeine intake, have shown similar inverse associations with T2DM risk [66–68]. Additionally, participants who consumed both ground coffee (filtered or espresso) and instant coffee exhibited a lower incidence of T2DM [52]. Given that coffee consumption in countries like the Netherlands is predominantly caffeinated, with a relatively small proportion of decaffeinated coffee drinkers, the impact of this limitation on our findings is likely minimal. Nonetheless, future studies with more detailed assessment of coffee types and brewing methods are warranted to clarify their potentially distinct effects on diabetes risk.

Fifth, we did not quantify specific bioactive compounds in coffee (such as chlorogenic acid or polyphenols), nor were we able to adjust for their intake from other dietary sources like fruits and vegetables. This is a relevant limitation, as the observed associations may be influenced by these compounds, and failing to control for them may introduce residual confounding. While we used total coffee intake (cups/day) as a pragmatic and standardized exposure measure, future research should aim to incorporate compound-level dietary data to refine mechanistic interpretations.

Lastly, while we adjusted for a wide range of potential confounders, residual confounding may still be significant, particularly among participants with high coffee consumption. Specifically, our adjustment for smoking was limited to smoking status (never, current, or former smoker) without accounting for factors such as smoking intensity and duration [69], which are known to influence T2DM risk. It is important to note that smoking may act as a suppressor rather than a confounder in this context, potentially influencing the observed associations between coffee consumption and glycemic outcomes among individuals who smoke. Although deep phenotyping allowed for adjustment of many variables, more detailed assessments of smoking behavior and other potential confounders may help improve the interpretation and reliability of these associations.

## Conclusion

In conclusion, no significant interaction was observed between coffee consumption and genetic susceptibility (PRS) for diabetes, suggesting that genetic predisposition may have a consistent influence regardless of coffee consumption levels. Importantly, our findings also showed an association between coffee consumption and the prevalence of prediabetes and T2DM that appeared to vary by consume level, potentially following a U-shaped pattern. However, these associations are population-specific and may not generalize. Further research is needed to explore gene-lifestyle interactions in diverse populations.

## Supplementary Information

Below is the link to the electronic supplementary material.

Supplementary Material 1

## Data Availability

The data used in this study are sourced from the Maastricht Study. However, access to these data is restricted, as they were utilized under a specific license for this research. Data requests can be made to the authors, who may provide access contingent upon a reasonable request and approval from the Maastricht Study management team.

## Abbreviations

BMI: Body Mass Index
CI: Confidence interval
CVD: Cardiovascular disease
DHD: Dutch healthy diet
FFQ: Food frequency questionnaire
GWAS: Genome-wide association study
HWE: Hardy-Weinberg equilibrium
HDL: Serum HDL cholesterol
Kcal: Kilocalories
LD: Linkage disequilibrium
LDL: Serum LDL cholesterol
MAF: Minor allele frequency
NGM: Normal glucose metabolism
OR: Odds ratio
PCA: Principal component analysis
PCs: Principal components
PRS: Polygenic risk score
QC: Quality control
SNPs: Single nucleotide polymorphisms
T2DM: Type 2 diabetes mellitus
