# Supplementary material for: Interaction between coffee consumption and polygenic risk score in relation to diabetes: insights from the Maastricht study
Source: Eur J Nutr. 2025 Aug 20;64(6):263. doi: 10.1007/s00394-025-03782-y (PMC12367902; doi:10.1007/s00394-025-03782-y)
Supplement: Supplementary file 1 — Supplementary Material 1 [file 394_2025_3782_MOESM1_ESM.docx]

***Yufeng Rao et al. Interaction Between Coffee Consumption and Polygenic Risk Score in Relation to Diabetes: Insights from The Maastricht Study***

**Supplementary Materials**

**Supplementary Fig. 1** Flowchart of study participants

**Supplementary Table 1** Association between coffee consumption, PRS and the prevalence of newly diagnosed diabetes (n = 5,193; OR (95% CI))

**Supplementary Fig. 2** Dose-response association between coffee consumption and the prevalence of newly diagnosed diabetes (n = 5,193)

**Supplementary Table 2** The interaction between PRS and coffee consumption and the prevalence of newly diagnosed diabetes (OR (95% CI))

**Supplementary Table 3** Association between coffee consumption, PRS and the prevalence of prediabetes/T2DM adjusted for recruitment year (n = 7,668; OR (95% CI))

**Abbreviations:** BMI, Body Mass Index; CI, Confidence Interval; CVD, Cardiovascular Disease; DHD, Dutch Healthy Diet; OR, Odds Ratio; PRS, Polygenic Risk Score;


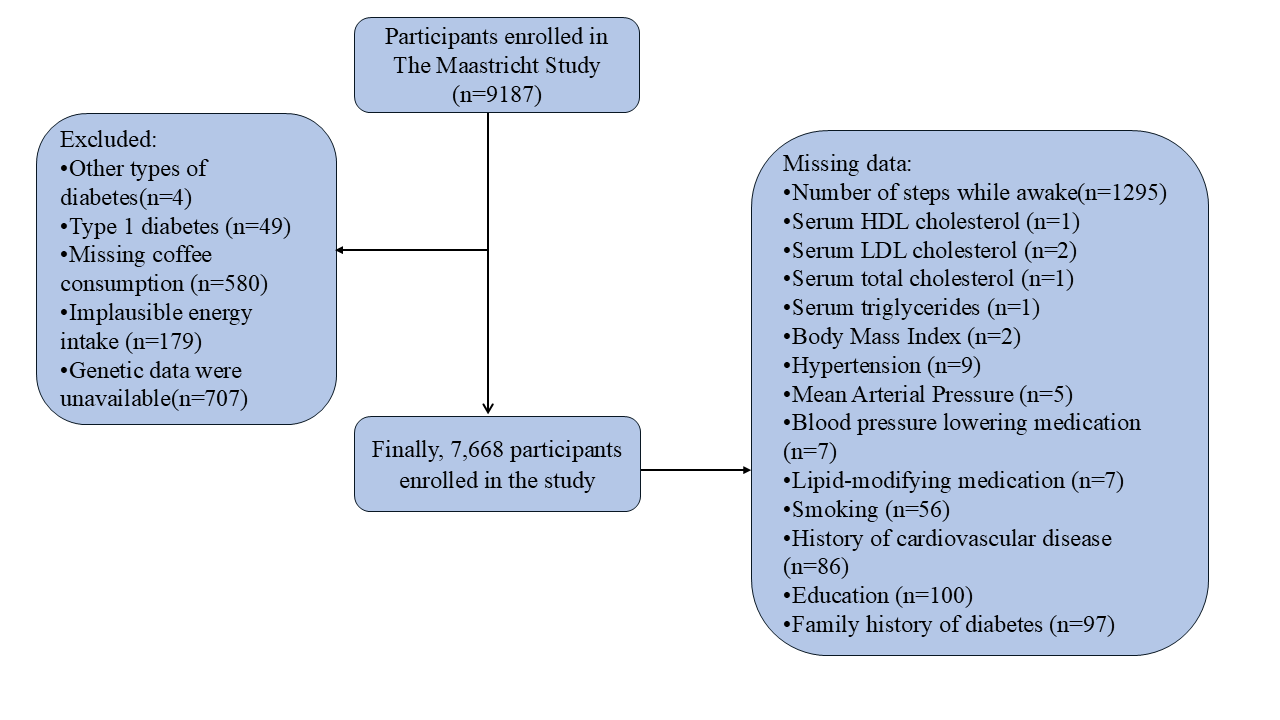


**Supplementary Fig. 1** Flowchart of study participants

**Supplementary Table 1** Association between coffee consumption, PRS and the prevalence of newly diagnosed diabetes (n = 5,193; OR (95% CI))

|  |  | **Newly diagnosed diabetes** | | |
| --- | --- | --- | --- | --- |
| **Predictor** |  | Model 1 | Model 2 | Model 3 |
| Coffee | Low | 1 | 1 | 1 |
|  | Medium | 0.91 (0.69, 1.20) | 0.86 (0.63, 1.19) | 0.88 (0.63, 1.23) |
|  | High | 0.86 (0.65, 1.15) | 0.69 (0.49, 0.98) | 0.76 (0.53, 1.10) |
| PRS | Low | 1 | 1 | 1 |
|  | Medium | 1.33 (0.99, 1.78) | 1.56 (1.11, 2.21) | 1.50 (1.05, 2.15) |
|  | High | 1.80 (1.35, 2.39) | 2.20 (1.57, 3.09) | 1.99 (1.40, 2.83) |

Model 1: was adjusted for age, sex, and educational level.

Model 2: was additionally adjusted for body mass index (BMI), alcohol consumption, smoking status, number of steps while awake, Dutch Healthy Diet (DHD), and energy intake.

Model 3: was additionally adjusted for cardiovascular disease (CVD), serum total cholesterol, mean arterial pressure, use of blood pressure lowering medication, use of lipid-modification medication, sugar consumption, and family history of diabetes.

Statistical data for different coffee groups (median [Q1, Q3], min-max): Low coffee group = 2.0 [0.7, 2.2], 0-2.6; Medium coffee group = 3.9 [3.0, 4.0], 2.8-4.0; High coffee group = 6.0 [5.2, 7.0], 4.3-16.9.

Statistical data for different PRS groups (median [Q1, Q3], min-max): Low PRS group = 1.4 [1.0, 1.6], -0.9-1.8; Medium PRS group = 2.2 [2.0, 2.3], 1.8-2.5; High PRS group = 3.0 [2.7, 3.3], 2.5-4.9.


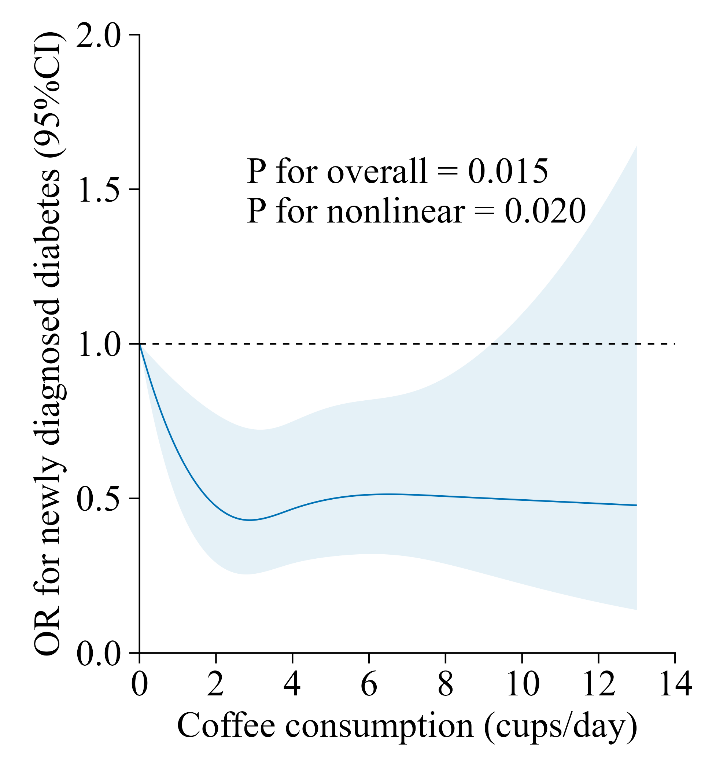


**Supplementary Fig. 2** Dose-response association between coffee consumption and the prevalence of newly diagnosed diabetes (n = 5,193)

The dose-response association between coffee consumption and the prevalence of newly diagnosed diabetes was modeled using restricted cubic spline with 4 knots placed at the 5th, 35th, 65th, 95th percentiles of coffee consumption. The reference was set at the 5th percentile, corresponding to 0 cups/day. Model was adjusted for age, sex, educational level, body mass index (BMI), alcohol consumption, smoking status, number of steps while awake, Dutch Healthy Diet (DHD), energy intake, cardiovascular disease (CVD), serum total cholesterol, mean arterial pressure, use of blood pressure lowering medication, use of lipid-modification medication, sugar consumption, and family history of diabetes. The solid blue line indicates ORs, the blue shaded area indicates 95% Cis. OR, odds ratio; CI, confidence interval.

**Supplementary Table 2** The interaction between PRS and coffee consumption and the prevalence of newly diagnosed diabetes (OR (95% CI))

| **Coffee** | **PRS** | **Newly diagnosed diabetes** | | |
| --- | --- | --- | --- | --- |
|  |  | Model 1 | Model 2 | Model 3 |
| Low  (n = 1,974) | Low | 1 | 1 | 1 |
|  | Medium | 1.51 (0.94, 2.41) | 1.53 (0.88, 2.66) | 1.50 (0.83,2.70) |
|  | High | 1.72 (1.08, 2.76) | 2.11 (1.23, 3.61) | 1.79 (1.00,3.19) |
| Medium  (n = 1,670) | Low | 1 | 1 | 1 |
|  | Medium | 1.68 (1.00, 2.82) | 2.12 (1.14, 3.95) | 1.83 (0.96, 3.46) |
|  | High | 2.61 (1.57, 4.33) | 3.43 (1.84, 6.37) | 3.32 (1.76, 6.26) |
| High  (n = 1,549) | Low | 1 | 1 | 1 |
|  | Medium | 0.89 (0.52, 1.53) | 1.14 (0.59, 2.22) | 1.09 (0.55, 2.19) |
|  | High | 1.29 (0.77, 2.15) | 1.56 (0.83, 2.94) | 1.36 (0.69, 2.67) |

Model 1: was adjusted for age, sex, and educational level.

Model 2: was additionally adjusted for body mass index (BMI), alcohol consumption, smoking status, number of steps while awake, Dutch Healthy Diet (DHD), and energy intake.

Model 3: was additionally adjusted for cardiovascular disease (CVD), serum total cholesterol, mean arterial pressure, use of blood pressure lowering medication, use of lipid-modification medication, sugar consumption, and family history of diabetes.

Statistical data for different coffee groups (median [Q1, Q3], min-max): Low coffee group = 2.0 [0.7, 2.2], 0-2.6; Medium coffee group = 3.9 [3.0, 4.0], 2.8-4.0; High coffee group = 6.0 [5.2, 7.0], 4.3-16.9.

Statistical data for different PRS groups (median [Q1, Q3], min-max): Low PRS group = 1.4 [1.0, 1.6], -0.9-1.8; Medium PRS group = 2.2 [2.0, 2.3], 1.8-2.5; High PRS group = 3.0 [2.7, 3.3], 2.5-4.9.

**Supplementary Table 3** Association between coffee consumption, PRS and the prevalence of prediabetes/T2DM adjusted for recruitment year (n = 7,668; OR (95% CI))

| **Predictor** |  | **Prediabetes** | | | **T2DM** | | |
| --- | --- | --- | --- | --- | --- | --- | --- |
|  |  | Model 1 | Model 2 | Model 3 | Model 1 | Model 2 | Model 3 |
| Coffee | Low | 1 | 1 | 1 | 1 | 1 | 1 |
|  | Medium | 0.76  (0.65, 0.89) | 0.80  (0.70, 0.91) | 0.80  (0.69, 0.92) | 0.79  (0.68, 0.92) | 0.83  (0.73, 0.94) | 0.80  (0.70, 0.91) |
|  | High | 0.88  (0.75, 1.04) | 0.84  (0.74, 0.97) | 0.83  (0.71, 0.96) | 0.95  (0.81, 1.10) | 0.87  (0.76, 0.99) | 0.79  (0.70, 0.90) |
| PRS | Low | 1 | 1 | 1 | 1 | 1 | 1 |
|  | Medium | 1.29  (1.09, 1.51) | 1.38  (1.19, 1.60) | 1.35  (1.15, 1.58) | 2.24  (1.90, 2.65) | 2.44  (2.16, 2.74) | 2.23  (1.98, 2.52) |
|  | High | 1.68  (1.43, 1.98) | 1.72  (1.48, 1.99) | 1.58  (1.35, 1.85) | 3.59  (3.05, 4.22) | 3.99  (3.56, 4.48) | 3.11  (2.76, 3.51) |

Model 1: was adjusted for age, sex, educational level, and recruitment period (early 2010-2014 vs. late 2015-2020).

Model 2: was additionally adjusted for body mass index (BMI), alcohol consumption, smoking status, number of steps while awake, Dutch Healthy Diet (DHD), and energy intake.

Model 3: was additionally adjusted for cardiovascular disease (CVD), serum total cholesterol, mean arterial pressure, use of blood pressure lowering medication, use of lipid-modification medication, sugar consumption, and family history of diabetes.

Statistical data for different coffee groups (median [Q1, Q3], min-max): Low coffee group = 2.0 [0.7, 2.2], 0-2.6; Medium coffee group = 3.9 [3.0, 4.0], 2.8-4.0; High coffee group = 6.0 [5.2, 7.0], 4.3-16.9.

Statistical data for different PRS groups (median [Q1, Q3], min-max): Low PRS group = 1.4 [1.0, 1.6], -0.9-1.8; Medium PRS group = 2.2 [2.0, 2.3], 1.8-2.5; High PRS group = 3.0 [2.7, 3.3], 2.5-4.9.
